# Increased spontaneous recombination in RNase H2-deficient cells arises from multiple contiguous rNMPs and not from single rNMP residues incorporated by DNA polymerase epsilon

**DOI:** 10.15698/mic2016.06.506

**Published:** 2016-05-15

**Authors:** Anastasiya Epshtein, Catherine J. Potenski, Hannah L. Klein

**Affiliations:** 1 Department of Biochemistry and Molecular Pharmacology, New York University School of Medicine, 550 First Avenue, New York, New York 10016, USA.; 2 Nature Publishing Group, One New York Plaza, New York, New York 10004, USA.

**Keywords:** RNase H2, recombination, rNMP, DNA polymerase

## Abstract

Ribonucleotides can become embedded in DNA from insertion by DNA polymerases, failure to remove Okazaki fragment primers, R-loops that can prime replication, and RNA/cDNA-mediated recombination. RNA:DNA hybrids are removed by RNase H enzymes. Single rNMPs in DNA are removed by RNase H2 and if they remain on the leading strand, can lead to mutagenesis in a Top1-dependent pathway. rNMPs in DNA can also stimulate genome instability, among which are homologous recombination gene conversion events. We previously found that, similar to the rNMP-stimulated mutagenesis, rNMP-stimulated recombination was also Top1-dependent. However, in contrast to mutagenesis, we report here that recombination is not stimulated by rNMPs incorporated by the replicative polymerase epsilon. Instead, recombination seems to be stimulated by multiple contiguous rNMPs, which may arise from R-loops or replication priming events.

## INTRODUCTION

In the last few years it has become apparent that the replicative polymerases misincorporate rNMP residues into DNA at rates that may become a significant burden to genome stability. It has been estimated that during replication about 15,000 rNMP become incorporated into the nuclear genome of the yeast *Saccharomyces cerevisiae* 1. These rNMP residues can distort the DNA helix and impede progression of RNA and DNA polymerase machineries. Additional sources of rNMPs in DNA may come from incomplete removal of the Okazaki fragment primers. The embedded rNMPs are removed quite efficiently by the nuclease RNase H2, which recognizes both single and multiple rNMP residues in a RNA:DNA hybrid conformation 2,3 or by RNase H1, which has specificity towards multiple contiguous rNMP residues of four or more paired with dNMPs 2. The process of removal of single rNMP residues during replication is termed ribonucleotide excision repair (RER) 4.

The RNase H1 ribonuclease acts primarily on R-loops, which differ in structure from rNMPs embedded in DNA. R-loops are formed during replication and have a RNA:DNA duplex strand opposite an unpaired single DNA strand. R-loops also form during high transcription 5,6,7,8, often the result of transcription replication collisions 6,9,10. Detection using an antibody against RNA:DNA hybrids has revealed the natural occurrence of these hybrids at highly transcribed genes, tRNA genes, Ty sequences, and telomeres 11,12.

High transcription, R-loop formation and transcription replication collisions are known to stimulate DNA break formation and recombination 5,8,13,14,15. High transcription can result in increased gene conversion 15, but it is not known whether this is associated with R-loop formation 13. R-loop formation results in increased recombination that can be reduced by over-expression of RNase H1 6,9,16, suggesting that persistent R-loops can provoke recombination-promoting lesions. Further evidence for a role for transcription and RNase H comes from studies on gross chromosomal rearrangements, which are reduced when RNase H1 is overexpressed in cells with altered transcription and chromatin structure 14. Recently, transcription-dependent R-loops have been shown to be able to initiate replication at regions other than replication origins and cause genome instability from endoreduplication and copy number changes 17.

In the absence of functional RNase H2 enzyme, rNMPs remain in DNA, as evidenced by the alkali sensitivity of genomic DNA 18. rNMPs on the leading strand can be cleaved by the action of Topoisomerase I (Top1) 19,20, generating a nick that can lead to further processing and signature deletion mutations in simple repeats 19,21.

In addition to deletion mutations, loss of RNase H2 function is characterized by hyper-recombination between directly repeated sequences 22, chromosome loss 23, and chromosome rearrangements 23. Indeed, a mutation in an RNase H2 subunit was recovered as a hyper-rec mutant in an early screen for such mutants 24. Studies on the susceptibility of hyper-recombination to Top1 action in an RNase H2-defective cell environment showed that similar to increased deletion mutation, hyper-recombination also sensitive to Top1 and is not stimulated in a Top1-defective strain. This finding promoted us to further explore the nature of the initiating lesion for hyper-recombination in the absence of RNase H2.

## RESULTS

### RNase H2 mutants have an increased recombination phenotype

In our collection of hyper-rec (*hpr*) mutants that we isolated on the basis of increased intrachromosomal recombination 24 was the *hpr4-1* mutant. We were intrigued by this mutant as it showed an increased rate of mitotic gene conversion, but did not deviate in the pattern of recombination events from wild type. Moreover, the mutant also had a mutator phenotype while displaying no DNA damage sensitivity, but was synthetically sick with mutants of the MRX complex. This suggested a defect in processing DNA break damage or a bypass pathway that could not process all DNA break damage. Subsequent studies identified *hpr4-1 *as an allele of *RNH202*, a subunit of yeast RNase H2 22.

**Figure 1:**
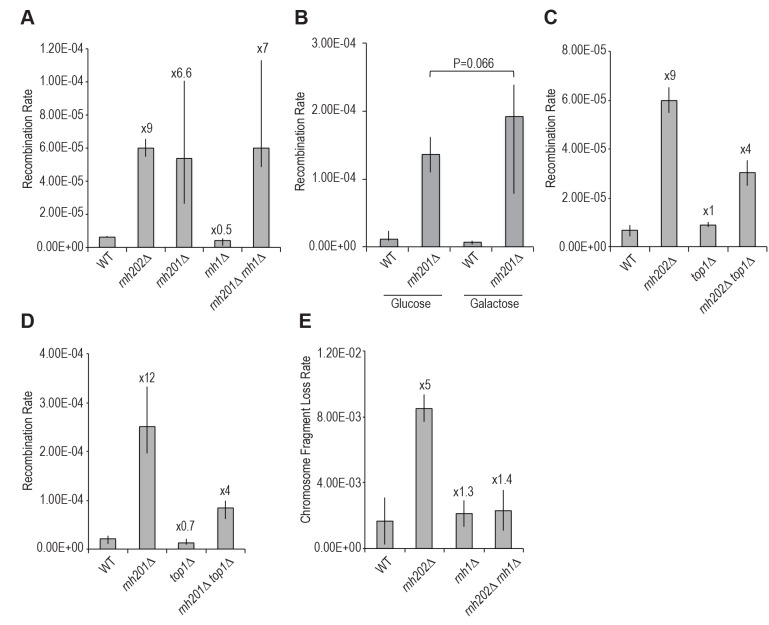
FIGURE 1: Intrachromosomal gene conversion is increased in RNase H2-defective cells. **(A)** Recombination rates of gene conversion are shown as median rates with 95% confidence limits (*n *= 18) in RNase H1 and RNase H2 mutant strains. The reporter is a duplication of *leu2 *genes at the *LEU2 *locus. **(B)** Effect of RNase H1 over-expression in intrachoromosomal gene conversion rates. Recombination rates of the *ade2 *duplication reporter with a Gal-Rnh1 plasmid are shown with 95% confidence limits (*n *= 12). **(C)** Effect of *TOP1 *deletion on the gene conversion recombination rate using the *leu2 *duplication reporter. The wild type (WT) and *rnh202*∆ rates are taken from panel A. **(D)** Recombination rates of gene conversion are shown as median rates with 95% confidence limits (*n *= 18) in RNase H2 mutant strains using a different recombination reporter consisting of a duplication of *ade2 *genes at the *ADE2 *locus. The effect of *TOP1 *deletion on this reporter is also shown. **(E)** Chromosome fragment loss in a haploid strain is shown. Median rates with standard deviations are displayed.

To further characterize the hyper-rec phenotype, we determined recombination rates in an *rnh202*Δ strain, in addition to rates from an *rnh201*Δ strain and an *rnh1*Δ strain. While the *rhn201 *and *rnh202 *mutants had similar elevated recombination rates, recombination in the *rnh1 *strain did not change from wild type (Figure 1A). Moreover, addition of the *rnh1 *mutation to an *rnh202 *strain did not enhance the *rnh202 *recombination rate (Figure 1A). Overexpression of RNase H1 did not reduce the increased recombination seen in an *rnh201* mutant (Figure 1B), further suggesting that the increased gene conversion does not arise from a substrate that accumulates in the absence of RNase H1.

Recent reports have found that using a global genome analysis of RNase H2-defective strains there is a widespread occurrence of recombination and loss of heterozygosity in diploid strains 25,26. As the increased mutation rates observed in *rnh2* mutants are dependent on Top1 18,19,21,22, and one report found that loss of heterozygosity crossing over and nonallelic recombination was Top1-dependent 25, we examined recombination in an *rnh202*Δ* top1*Δ strain. As previously reported, hyper-recombination in *rnh202*Δ requires Top1 activity 22 (Figure 1C) and we observed a similar Top1-dependence using the second recombination reporter (Figure 1D). We used two different recombination reporters because we wanted to verify that the increased recombination, gene conversion, was not reporter specific or genome location specific. The genome-wide studies of recombination increase have focused on loss of heterozygosity and reciprocal crossing over in diploids 25,26 whereas in this case we have examined gene conversion in haploids. In all cases, loss of RNase H2 has resulted in increased recombination and genome instability.

### Increased genome instability is observed in RNase H2-defective strains

Our early studies revealed that the *rnh202*Δ mutant has additional genome instability phenotypes of chromosome loss that are also observed in a mouse RNase H2 knockout line 23 as well as in genome studies of yeast RNase H2-defective cells 25,26. To further measure spontaneous genome instability, we determined loss of a chromosome fragment in haploid *rnh202*Δ strains (Figure 1E). Loss of RNase H2 function greatly stimulated genome instability, while loss of RNase H1 did not result in an increase. Curiously in the double RNase H mutant the *rnh1*∆ rate was observed, suggesting that processing of *rnh2*-lesions by RNase H1 leads to chromosome instability.

### Increased recombination is stimulated by high transcription

**Figure 2:**
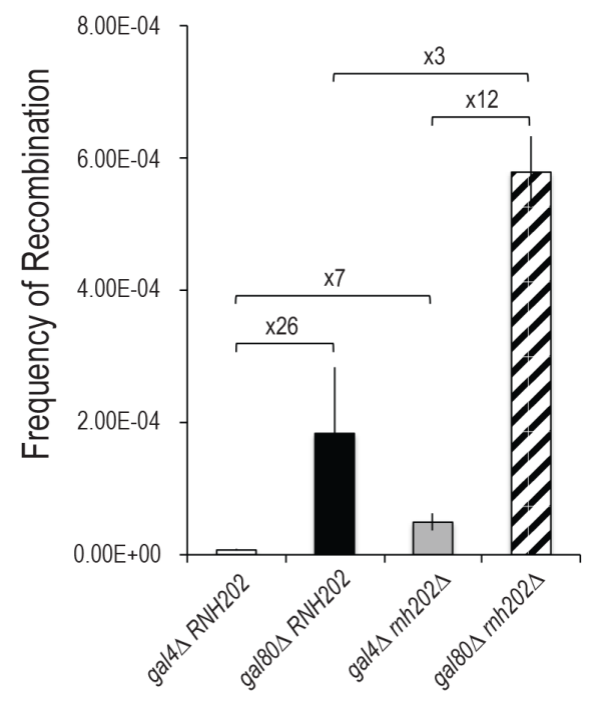
FIGURE 2: Transcription stimulation of recombination is independent of *rnh202 *stimulation of recombination. Recombination between duplicated *gal10 *genes at the *GAL10 *locus as a function of transcription status is shown as median rates with standard deviations (*n *= 27). Low transcription occurs in *gal4*∆ strains while high transcription occurs in *gal80*∆ strains. Fold differences between indicated genotypes are shown.

Recombination can be stimulated by high transcription 13,15. To determine if recombination in an *rnh202*Δ strain was linked to transcription, we examined recombination in a reporter under low and high transcription conditions. The reporter was a duplication of *GAL10 *genes, each with a different mutation, in strains with low transcription (*gal4*) and high transcription (*gal80*) levels of the *GAL10 *genes (Figure 2). In a wild type strain, recombination was stimulated 26-fold, as previously reported 15. Similarly, in an *rnh202*Δ strain recombination was stimulated under high transcription conditions. The increase was more than additive, suggesting that in a recombination permissive state, that is, under high transcription, loss of RNase H2 increases transcription-stimulated recombination.

### Increased rNMP incorporation into DNA does not alter recombination rates

**Figure 3:**
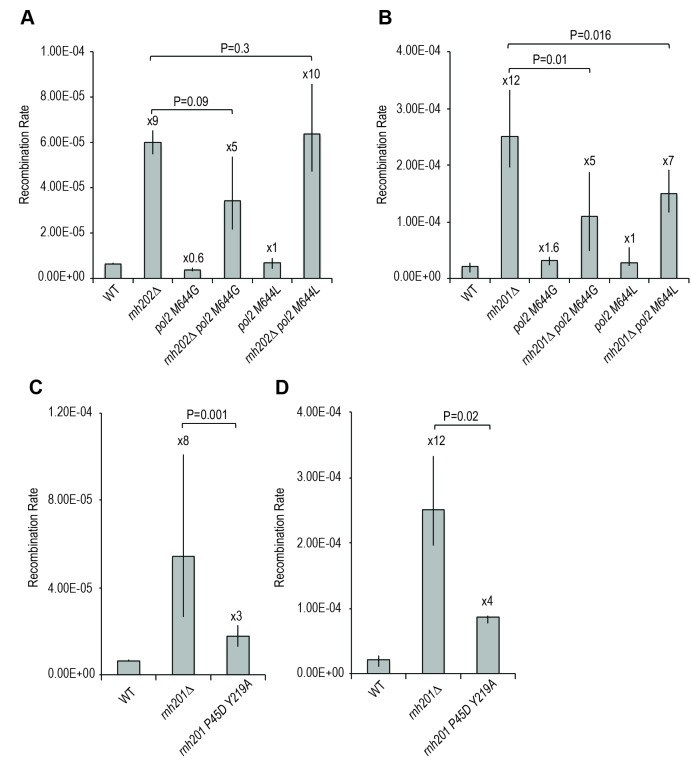
FIGURE 3: Effect of DNA polymerase epsilon mutants and RNase H2 mutants on recombination rates. **(A)** Recombination rates using the *leu2 *duplication reporter in strains with increased (*pol2 M644G) *or decreased (*pol2 M644L*) rNMP incorporation are shown. Median rates with 95% confidence limits (*n *= 18) are presented. **(B)** Recombination rates using the *ade2 *duplication reporter in strains with increased (*pol2 M644G) *or decreased (*pol2 M644L*) rNMP incorporation are shown. Median rates with 95% confidence limits (*n *= 18) are presented. **(C)** Recombination rates in an *rnh201 P45D Y 219A *strain are shown using the *leu2 *duplication reporter are shown. Median rates with 95% confidence limits (*n *= 18) are presented. **(D)** Recombination rates in an *rnh201 P45D Y 219A *strain using the *ade2 *duplication reporter are shown. Median rates with 95% confidence limits (*n *= 18) are presented. The wild type (WT) and *rnh201∆ *rates are taken from panel B.

rNMP residues can become embedded in DNA through the action of the replicative polymerases. These can stimulate mutation as use of a DNA polymerase epsilon allele, *pol2-M644G*, that has reduced sugar specificity and allows 10-fold higher incorporation of rNMPs into DNA, also increases deletion mutations 18, dependent on Top1 activity 19,21,27. Similarly, the *pol2-M644L *allele, which reduces rNMP incorporation, also reduces the rate of deletion mutations. As we have found that the *rnh202*Δ hyper-recombination is dependent on Top1 function, we determined recombination rates in *rnh202*Δ* pol2-M644G *and *rnh202*Δ* pol2-M644L *strains (Figure 3A-B). Unexpectedly, we found that the polymerase epsilon allele *pol2-M644G* did not increase the recombination rates, as would be expected, and the polymerase epsilon allele *pol2-M644L* did not reduce the rate using the *leu2 *duplication reporter and only slightly reduced the rate with the *ade2 *duplication reporter.

We were concerned that the *POL2 *mutants could affect the growth rate of cells, particularly in S phase, and that this by itself could provide more opportunity for DNA damage and recombination and hence we determined the doubling times of *rnh202 POL2, rnh202 pol2-M644G *and *rnh202 pol2-M644L *strains. We found that the presence of the *pol2-M644L *mutation did not alter the growth rate of *rnh202∆ *cells, further suggesting that the presence of misincorporated rNMP residues on the leading strand does not account for the increased recombination phenotype. The *rnh202 pol2-M644G* had a slightly reduced growth rate but this did not account for unaltered recombination rate.

### Separation of function *rnh201 *mutant

Eukaryotic RNase H2 enzymes, including the yeast RNase H2 heterotrimer, can cleave at single rNMPs in DNA but also can cleave at multiple consecutive rNMPs, recognizing the RpR transition bound to DNA 3. The RpR/DNA structure is termed an R-loop 3. Using structural information from bacterial RNase H enzymes with different specificities against RpR/DNA and RpD/DNA hybrids, a separation of function mutant of *RNH201*, the catalytic subunit of yeast RNase H2 was constructed 3. This mutant is completely defective in processing RpD/DNA hybrids, the result of single rNMP residues in DNA, but is able to process RpR/DNA hybrids or R-loops with reasonable efficiency 3. We used this mutant to assess recombination rates, to determine which substrate stimulated hyper-recombination. We observed that retention of the R-loop processing activity was sufficient to significantly reduce recombination rates (Figure 3C-D), suggesting that recombination is stimulated by cleavage at RpR/DNA sites, consistent with our results using mutant *POL2 *alleles that did not greatly affect *rnh2 *recombination rates.

## DISCUSSION

Hyper-recombination is caused by either creating more recombination-initiating lesions or by changing how a lesion is repaired, from a non-recombination gap filling reaction to a homologous recombination mode 28. Many hyper-rec mutants fall into the first class, examples being defective components of the DNA replication apparatus, which can leave nicks in DNA that are processed into recombination-initiating breaks. Examples of the second class are mutants that fail to provide anti-recombination activity, such as mutation of the Srs2 DNA helicase such that it fails to removes Rad51 nucleofilaments that thereby prevent recombination. 

In the case of mutants in the RNase H2 complex, these appear to be of the first class in that rNMPs remain in DNA and are ultimately processed by enzymes that form nicks. One possibility for nick formation is through Top1 action, which is known to cleave at rNMPs remaining on the leading strand 20. As the hyper-recombination phenotype of the *rnh2 *mutants is dependent on Top1 function, it is logical to infer that Top1 cleaves at such rNMPs on the leading strand and these may ultimately be processed to a double strand break or a single strand gap that can initiate recombination. The study of Conover *et al.* 25 examined genome instability in diploid yeast, measuring loss of heterozygosity through crossing over and nonallellic homologous recombination. Both of these genome instability events were reduced in a mutant *pol2 *strain that incorporated fewer rNTPs and increased in a mutant *pol2 *strain that incorporated more rNTPs. Moreover, the stimulation in genome instability was dependent on Topoisomerase 1. These results are consistent with leading strand rNMPs and subsequent processing by Topoisomerase 1 to create recombinogenic lesions.

In contrast, the study of O’Connell* et al. *26, measuring global loss of heterozygosity events in diploid yeast and reciprocal crossing over, did not observe any reduction in instability rate in mutant *pol2 *strain that incorporated fewer rNTPs. Moreover, the instability rate of an *rnh201*∆ diploid strain was increased by a *rnh1*∆ mutation, suggesting that the recombinogenic lesions arise from multiple contiguous rNMP residues or R-loops.

Our results are more in line with those from the O’Connell group, but we have not observed any stimulation of recombination by an *rnh1*∆ mutation or reduction by over-expression of *RNH1. *We have examined intrachromatid or intersister chromatid gene conversion, of short gene conversion tracts with a maximum length of 510 nucleotides in haploid strains. The cell cycle timing of recombination and regulation in haploid intrachromosomal recombination may differ from the global diploid loss of heterozygosity studies.

The separation of function *rnh201 *mutant has provided insight into the origin of recombination by rNMPs. The mutant is completely deficient in the removal of single rNMP residues incorporated into DNA during DNA replication 3. However, it retains *in vitro* activity against a tract of six rNMP residues in a RNA:DNA hybrid, suggesting that it can cleave at RpR/DNA sites but not at RpD/DNA sites. Although the *in vitro* activity of the mutant RNase H2 is reduced from wild type 3, it is sufficient for *in vivo* rescue of *sgs1*∆ *rnh201*∆ synthetic growth defect. We have found that it is sufficient to suppress most of the hyper-recombination seen in an *rnh20*Δ mutant. The residual increased recombination could arise from a partial loss of activity against RpR/DNA sites *in vivo*, as suggested by the partial *in vitro* activity of the mutant 3, or it could reflect that some intrachromosomal gene conversion events are stimulated by single rNMPs in DNA, but they represent a fraction of total events that cannot be detected as altered by the *pol2 *mutants. The *in vivo* origin of the substrate recognized by this *rnh201 *allele is not clear, but could result from transcription replication conflicts at the replication fork, producing R-loops that eventually result in a recombination lesion in the absence of RNase H2. However, recombination levels are not further augmented by loss of RNase H1 in the recombination reporters we have used, and overexpression of RNase H1 does not reduce recombination levels in an *rnh201*Δ mutant. These observations suggest that the RNA:DNA hybrid that stimulates recombination is not a conventional R-loop. Indeed, the recombination-initiating lesion might not be an R-loop arising from transcription, but could instead come from ribonucleotides in DNA that remain in the lagging strand. 

As further evidence of the complexity of genome destabilization from embedded rNMPs, we found that chromosome fragment loss was elevated by loss of RNase H2, but it appears that the action of RNase H1 on the ensuing lesions causes the chromosome fragment loss. We suggest that recombination arises from breaks at the replication fork that occasionally arise during replication. These may come from replication transcription collisions or from consequences of stalled replication. Alternatively, rNMPs on the lagging strand may cause stalling of the replication machinery in the next replication cycle, leading to a double strand break that can initiate recombination. Experiments to address the nature of the RNA:DNA intermediate formed in the absence of RNase H2 activity is currently under investigation. 

## MATERIALS AND METHODS

### Strains

All strains used in this study are listed in Table S1.

### Recombination and chromosome loss rates

Recombination rates were performed using the *leu2-ecoRI::URA3::leu2-bstEII *system as described 24 or the *ade2-n::TRP1::ade2-1-Sce1 *as described 29. Strains were streaked on YPD medium or selective medium to obtain single colonies. At least 12 or 18 independent cultures with a minimum of two isolates per genotype were used to determine rates and 95% confidence limits 30. The transcription recombination reporter *gal10-kpnI::URA3::gal10-3*’Δ was used as described 15. Chromosome fragment loss was determined as described 31, using at least three independent strains for each genotype. Recombination rates with Rnh1 over-expression was performed using *ade2-n::TRP1::ade2-1-Sce1 *strains transformed with pRS416-GAL-RNH1(URA3). At least two strains of each genotype with the plasmid were streaked to SC-URA glucose medium. After growth, 12 independent colonies were used to inoculate liquid cultures of SC-URA glucose medium and 12 independent colonies were used to inoculate liquid cultures of SC-URA galactose medium. After growth for 24 hours, cells were collected by centrifugation, washed, resuspended in 1 ml water, and used at appropriate dilutions for fluctuation test analyses.

## SUPPLEMENTAL MATERIAL

Click here for supplemental data file.

All supplemental data for this article are also available online at http://microbialcell.com/researcharticles/increased-spontaneous-recombination-in-rnase-h2-deficient-cells-arises-from-multiple-contiguous-rnmps-and-not-from-single-rnmp-residues-incorporated-by-dna-polymerase-epsilon/.
