# Supplementary material for: Increased spontaneous recombination in RNase H2-deficient cells arises from multiple contiguous rNMPs and not from single rNMP residues incorporated by DNA polymerase epsilon
Source: Microb Cell. 2016 May 15;3(6):248–54. doi: 10.15698/mic2016.06.506 (PMC5305187; doi:10.15698/mic2016.06.506)
Supplement: Supplementary file 1 [file mic-03-248-s01.pdf]

Table S1. Yeast strains

579-10A	***MATa ade2-1 can1-100 his3-11,15 leu2-3,112 trp1-1 ura3-1 RAD5+***
660-2A	*MATalpha leu2-ecoRI::URA3::leu2-bstEII*
660-2B	*MATalpha leu2-ecoRI::URA3::leu2-bstEII*
3327-1A	*MATalpha rnh202::KanMX4 leu2-ecoRI::URA3::leu2-bstEII*
3327-3C	*MATa rnh202::KanMX4 leu2-ecoRI::URA3::leu2-bstEII*
3329-1B	*MATalpha rnh201::NAT leu2-ecoRI::URA3::leu2-bstEII*
3329-18C	*MATa rnh201::NAT leu2-ecoRI::URA3::leu2-bstEII*
2399-7B	*MATa rnh1::hphMX4 leu2-ecoRI::URA3::leu2-bstEII*
2399-3C	*MATalpha rnh1::hphMX4 leu2-ecoRI::URA3::leu2-bstEII*
3343-31B	*MATa rnh201::NAT rnh1::hphMX4 leu2-ecoRI::URA3::leu2-bstEII*
3343-34B	*MATalpha rnh201::NAT rnh1::hphMX4 leu2-ecoRI::URA3::leu2-bstEII*
LSY1519-1D	*MATalpha ade2-n::TRP1::ade2-I-SceI+/AatII*
3342-4	*MATalpha rnh201::KanMX4 ade2-n::TRP1::ade2-I-SceI+/AatII*
3342-14	*MATalpha rnh201::KanMX4 ade2-n::TRP1::ade2-I-SceI+/AatII*
LSY1598	*MATa CF(CEN URA3 SUP11 ARS)*
2899-1D	*MAT rnh202::KanMX4 CF(CEN URA3 SUP11 ARS)*
2899-4B	*MAT rnh202::KanMX4 CF(CEN URA3 SUP11 ARS)*
2358-4B	*MATa rnh202::KanMX4 ADE2 CAN1*
2359-2A	*MATalpha rnh202::KanMX4 hom3-10*
3330-2A	*MATalpha rnh201 P45D Y219A leu2-ecoRI::URA3::leu2-bstEII*
3330-4C	*Mata rnh201 P45D Y219A leu2-ecoRI::URA3::leu2-bstEII*
3350-4B	*MATalpha rnh201 P45D Y912A ade2-n::TRP1::ade2-I-SceI+/AatII*
3350-6B	*MATa rnh201 P45D Y219A ade2-n::TRP1::ade2-I-SceI+/AatII*
3353-3B	*MATalpha pol2 M644G ade2-n::TRP1::ade2-I-SceI+/AatII*
3353-10A	*MATalpha pol2 M644G ade2-n::TRP1::ade2-I-SceI+/AatII*
3353-1C	*MATa rnh201::NAT pol2 M644G ade2-n::TRP1::ade2-I-SceI+/AatII*
3353-10D	*MATa rnh201::NAT pol2 M644G ade2-n::TRP1::ade2-I-SceI+/AatII*
3354-1A	*MATalpha pol2 M644L ade2-n::TRP1::ade2-I-SceI+/AatII*
3354-4D	*MATa pol2 M644L ade2-n::TRP1::ade2-I-SceI+/AatII*
3354-3A	*MATalpha rnh201::NAT pol2 M644L ade2-n::TRP1::ade2-I-SceI+/AatII*
3354-3B	*MATa rnh201::NAT pol2 M644L ade2-n::TRP1::ade2-I-SceI+/AatII*
3327-3B	*MATa pol2 M644G leu2-ecoRI::URA3::leu2-bstEII*
3327-8D	*MATalpha pol2 M644G leu2-ecoRI::URA3::leu2-bstEII*
3327-3A	*MATalpha rnh202::KanMX4 pol2 M644G leu2-ecoRI::URA3::leu2-bstEII*
3327-4B	*MATa rnh202::KanMX4 pol2 M644G leu2-ecoRI::URA3::leu2-bstEII*
3284-5C	*MATa pol2 M644L leu2-ecoRI::URA3::leu2-bstEII*
3284-8D	*MATalpha pol2 M644L leu2-ecoRI::URA3::leu2-bstEII*
3284-1B	*MATalpha rnh202::KanMX4 pol2 M644L leu2-ecoRI::URA3::leu2-bstEII*
3284-2A	*MATalpha rnh202::KanMX4 pol2 M644L leu2-ecoRI::URA3::leu2-bstEII*
2654-4D	*MATa gal80::LEU2 gal10-KpnI::URA3::gal10-3'Δ*
2654-8B	*MATalpha gal80::LEU2 gal10-KpnI::URA3::gal10-3'Δ*
2662-8C	*MATalpha rnh202::KANMX4gal80::LEU2 gal10-KpnI::URA3::gal10-3'Δ*
2655-15A	*MATalpha rnh202::KANMX4gal4::LEU2 gal10-KpnI::URA3::gal10-3'Δ*
HFY1125-11B	*MATalpha gal4::LEU2 gal10-KpnI::URA3::gal10-3'Δ*
HFY1125-12B	*MATa gal4::LEU2 gal10-KpnI::URA3::gal10-3'Δ*
3384-1A	*MATa rnh202::KanMX4 rnh1::hphMX4 CF(CEN URA3 SUP11 ARS)*
3384-3B	*MATalpha rnh202::KanMX4 rnh1::hphMX4 CF(CEN URA3 SUP11 ARS)*
3384-4B	*MATa rnh202::KanMX4 rnh1::hphMX4 CF(CEN URA3 SUP11 ARS)*
3384-2C	*MATalpha rnh1::hphMX4 CF(CEN URA3 SUP11 ARS)*
3384-4C	*MATalpha rnh1::hphMX4 CF(CEN URA3 SUP11 ARS)*
3384-16D	*MATa rnh1::hphMX4 CF(CEN URA3 SUP11 ARS)*
